# Bedside PDA ligation in premature infants less than 28 weeks and 1000 grams

**DOI:** 10.1186/s13019-016-0539-3

**Published:** 2016-10-04

**Authors:** Mustafa Kemal Avsar, Tolga Demir, Cem Celiksular, Cenap Zeybek

**Affiliations:** 1 Medicana International Istanbul Hospital Department of Cardiovascular Surgery, Istanbul, Turkey; 2 Kolan International Hospital Department of Cardiovascular Surgery, Istanbul, Turkey; 3 Medicana International Istanbul Hospital Depratment of Anesthesiology, Istanbul, Turkey; 4 Medicana International Istanbul Hospital Department of Pediatric Cardiology, Istanbul, Turkey

**Keywords:** Bedside surgery, Patent ductus arteriosus, Premature infant

## Abstract

**Background:**

PDA(Patent ductus arteriosus) is a common and clinically important condition which is presented with a number of hemodynamic and respiratory problems such as intraventricular hemorrhage, pulmonary hemorrhage and necrotizing enterocolitis due to increased pulmonary blood flow and stealing from systemic circulation. The incidence of PDA among the infants that were born before the 28th gestational week is as high as 70 %; and spontaneous closure rates in very-low-birth-weight premature neonates(VLBWPN) is around 34 %. The onset, duration, and repeat number of consecutive courses of the prostaglandin synthesis inhibitor medication for PDA closure are still issues of debate. Bed-side PDA closure is a safe surgical procedure in both mature and premature babies. Here we aim to retrospectively present our 26 cases which were less than 28 weeks and 1000 grams that underwent bed-side PDA ligation.

**Methods:**

This retrospective study included 26 VLBWPN with PDA that underwent bed-side ligation between 2012 and 2015. Babies were born before the 28th gestational week (23–27 weeks) and less than 1000 grams (489–970 gr). Of the 26, 15 were female and 11 were male. Indomethacin was administered to all of the cases as the medical closing agent. The medication was stopped due to unwanted effects in 6 cases. All of the patients took medical treatment before surgery.

**Results:**

No surgical mortality occurred during our study. One case of pneumothorax was recorded as late surgical complication. Five of the 26 patients were lost, and the most common cause of mortality was sepsis (in 3 cases). The remaining 21 cases were discharged on days 86–238. The follow-up periods of the patients were 2 moths - 3 years. The most frequent problems encountered after discharge was chronic lung problems.

**Conclusions:**

Bed side PDA ligation surgery in the ICU is a safe method for VLBWPN with clinically significant PDA.

## Background

Patent ductus arteriosus (PDA) is a serious condition of premature low-birth-weight infants. The important consequences of increased pulmonary blood-flow and stealing from systemic circulation due to left-to-right shunt in PDA include numerous hemodynamic and respiratory problems, such as intraventricular hemorrhage, pulmonary hemorrhage, pulmonary edema, necrotizing enterocolitis, retinopathy, decreased renal functions and chronic lung disease [1]. The incidence of PDA among premature babies less than 28 weeks and 1000 grams is as high as 70 %, and the rate of spontaneous closure in VLBWPN is about 34 %. The patency of arterial duct in prematurely born is brought about by several factors, including presence of lesser ductal medial muscles, relative oxygen hyposensitivity of the immature tissues, and increased sensitivity to prostaglandins [2].

To achieve ductal closure, neonatologists administer several treatment approaches in preterm infants with symptomatic PDA, such as cautious fluid replacement, diuretics, and prostaglandin synthesis inhibitors like ibuprofen and indomethacin. In the cases of contraindications for, or failure of medical treatment, surgical PDA ligation may be performed [3]. Timing of surgical intervention is still an issue of debate. Ko et al. advocate that ligation should be the treatment of choice for symptomatic PDA because of the increased risks brought with indomethacin treatment and prolonged intubation in VLBWPN [4].

The pros and cons of bedside vs. operation room interventions were discussed in several literatures. Low-birth-weight premature infants are usually intubated, their thermoregulation systems are sensitive, and they need numerous monitorization and intravenous infusion implementations. Thus to eliminate the possible risks of transportation, many advanced institutions now prefer bedside PDA ligation [4–6].

Here, not without citing the relevant literature, we aim to present our bed-side PDA ligation experience in premature infants less than 28 weeks and under 1000 grams with PDA, and related hemodynamic and pulmonary problems, in whom failure or complications of medical treatment had been encountered.

## Methods

This study was conducted retrospectively, and included a total of 26 VLBWPN, who underwent bedside PDA ligation between 2012 and 2015. Their birth weights were less than 1000 grams (480–970 gr), and the gestational ages at birth were less than 28 weeks (23–28 weeks).

Pre- and post-operative transthoracic echocardiography studies were performed by pediatric cardiologists. All infants were on mechanical ventilators, had cardiac failure, and received medication for cardiac failure. Criteria of clinically significant PDA were as follows: ductal diameter being above 1.5 mm, LA(left atrium)/Ao(aorta) ratio being above 1.4, presence of left-to-right shunt, reversal of end-diastolic blood flow in the aorta, and poor cardiac functions.

One indomethacin course included 0.2 mg/kg indomethacin three times every 12 h. Following a single course of indomethacin, PDA closure was evaluated by echocardiography. The cases without closure were given three additional courses of indomethacin. Side effects of indomethacin were present in 6 cases, which thus did not receive additional indomethacin medication after the first course. Those cases showed one or more of the following conditions: decreased urinary output (<0.5 ml/kg/h), high serum urea levels (>30 mg/dl), thrombocytopenia (<60,000/mm^3^), necrotizing enterocolitis, gastrointestinal bleeding, and intraventricular bleeding.

The bedside surgery team consisted of one cardiac surgeon, one anesthesiologist, one neonatal intensive care specialist, one scrub nurse, and one circulating nurse. Operations were performed in neonatal incubators with removable side walls and roofs, which was important during the intervention. Head lamps and portable lamps provided the illumination. The room was heated to 37 C^o^ preoperatively, and the head and extremities were covered to reduce heat loss. Following the anesthesia, skin antisepsis was achieved appropriately with iodine solution, and soaking was avoided. The skin was covered with drape and limited left posterolateral thoracotomy was performed. The lungs were retracted anteriorly, and PDA was explored. PDA was ligatured with two 2-0 silk sutures using double ligation transfixion technique. One 8 FR drainage tube was placed in all cases. The ribs were adducted with 2-0 vicryl. The skin was closed separately with 5-0 prolene mattress sutures. No perioperative complication was encountered.

## Results

The follow up duration of the patients were between 2 months and 3 years. Medical closure had been tried with one or more courses of ibuprofen of indomethacin in all of the patients. The cases in which either PDA was still open or medical treatment ceased due to complications underwent surgery. No patient was operated without prior medical treatment.

Demographical characteristics of the patients are summarized in Table 1.

**Table 1:** Demographical characteristics of the patients are summarized

Characteristic	*n* = 26
Birth weight range	510–960
Gestational age weeks	23–28
Female	15 (57 %)
Respiratory distress syndrome	26 (100 %)
Inotropes	22 (84.6 %)
Mortality	5 (19.2 %)
Sepsis	20 (76.9 %)
Severe intraventricular hemorrhage	10 (38.4 %)
Necrotizing enterocolitis	7 (26.9 %)
Severe renal dysfunction	5 (19.2 %)
Retinopathy of prematurity	8 (30.7 %)
Another cardiac pathology	6 (23.0 %)
Chronic lung disease	16 (61.5 %)
Time on ventilator; days, minimum-maximum	1–181
Time to discharge; days, minimum-maximum	40–238

Inotropes were used in the cases with myocardial dysfunction due to PDA and/or hypotension. The term sepsis was used only for the cases confirmed by positive blood cultures. The babies with grade 3–4 intraventricular bleeding were considered as severe intraventricular hemorrhage. Modified Bell criteria were used for the definition of necrotizing enterocolitis, and the cases being stage 2A or above were included in our analysis. Severe renal dysfunction was defined as having a blood urea nitrogen level of >150 μM/L, and having a urine output of <0.5 ml/kg/h during the last 12 h. Six of our patients had another cardiac pathology (2 patients with complete atrioventricular canal defect, 2 patients with atrial septal defect, 2 patient with ventricular septal defect). Patent foramen ovale was not included in this group. All of the patients were intubated before the operation, thus preoperative intubation periods are not represented in our data. Similarly all of the babies had cardiac failure either compensated, or decompensated, which is also not included in our data on Table 1. PDA diameters, mortality data, operation times, and body weights on the operation day are shown separately on Table 2.

**Table 2:** PDA diameters, mortality data, operation times, and body weights on the operation day are shown separately

Patient	Gestational age (weeks)	Operation weight (gram)	PDA size (mm)	Ligation age (days)	Duration of hospital stay (days)	Death
1^st^	25	580	3,1	10	150	
2^nd^	27	773	3,5	17	120	
3^rd^	27	752	2,3	37	141	
4^th^	24	509	1,6	15	168	
5^th^	26	624	2,7	20	40	Yes
6^th^	25	571	2,2	18	148	
7^th^	27	727	2,1	29	108	
8^th^	23	510	2,4	13	238	
9^th^	27	970	1,8	38	82	
10^th^	26	693	1,6	18	98	
11^th^	25	650	2,4	16	181	Yes
12^th^	26	715	2,5	9	86	
13^th^	27	876	3,4	12	94	
14^th^	27	715	3,1	15	68	Yes
15^th^	26	784	2,9	20	119	
16^th^	25	532	3,3	10	177	
17^th^	25	613	2,3	16	155	
18^th^	24	521	1,8	9	114	Yes
19^th^	27	810	2,6	25	112	
20^th^	26	603	2,7	21	138	
21^st^	26	697	2,2	30	141	Yes
22^nd^	27	708	3,2	7	163	
23th	24	489	2,4	19	212	
24^th^	26	686	2,8	30	114	
25^th^	27	786	2,9	16	118	
26^th^	27	723	3,4	8	197	

Only single courses of indomethacin were administered to the patients 1, 12, 16, 18, 22, and 26; and early PDA ligation operations were performed. The remaining cases were given three courses of indomethacin. Only PDA diameters are shown here, leaving out other relevant PDA criteria. Except five, no postoperative mortality was observed among the 26 patients that underwent bedside PDA ligation. Causes of mortality were demonstrated on Table 3.

**Table 3:** Causes of mortality among the babies that underwent PDA ligation

	Gender	Weight	GW	PC	OD	SAS (day)	COD
1	F	624	26	ARF, ICH	20	20	HE
2	F	715	27	ICH + PH	15	53	Sepsis
3	F	521	24	ARF	9	105	CLD,RF
4	M	650	25	T, Fever, L	16	167	Sepsis + HE
5	F	697	26	ICH + NEC	30	111	Sepsis

*GW* gestational week; *PC* preoperative condition; *OD* operation date; *PH* pulmonary hemorrhage; *T* thrombocytopenia; *ICH* intracranial hemorrhage; *RF* respiratory failure; *SAS* survival after surgery; *COD* cause of death; *ARF* acute renal failure; *NEC* necrotizing enterocolitis; *L* leukocytosis; *CLD* chronic lung disease; *HE* hypoxic encephalopathy

Two of the 5 cases with mortality had hypoxic encephalopathy and had been resuscitated for cardiac arrest several times during the preoperative period. Postoperative surgical complication was observed only in one patient, who developed pneumothorax. Twenty-one of the 26 cases that had been operated bedside were discharged on days 86–238. The most common problems encountered after the discharge were lung related.

## Discussion

Patent ductus arteriosus, which leads to significant hemodynamic, pulmonary, gastrointestinal, cerebrovascular, and retinal problems, can be medically or surgically treated; although, there has been an ongoing debate on which method should be preferred.

PDA ligation is generally performed in the cases in which indomethacin or ibuprofen medication is unsuccessful or contraindicated. However, studies show that PDA closure rates with medical treatment are still low in VLBWPN. In the study by Trust et al., which included infants under 800 g, the failure rates for PDA closure with indomethacin was found to be as high as 40–50 % [7]. In addition, indomethacin treatment in VLBWPN has numerous side effects such as necrotizing enterocolitis, bowel perforation, pulmonary edema and bleeding, retinopathy, thrombocytopenia, and decrease in renal functions [8]. Also there is a risk of reopening of the medically closed ductus, after the treatment [9].

Ibuprofen is as effective as indomethacin in PDA closure, with lower side effects on intestinal, renal, and cerebral blood flow. However, this drug deteriorates the renal functions in premature infants less than 26 weeks more prominently [10]. We chose indomethacin for medical closure of PDA in our study. Upon the side effects encountered, we stopped the medical treatment and performed surgery in 4 of our cases. The most common side effects were thrombocytopenia, necrotizing enterocolitis, and decreased renal functions.

There are still arguments about the potential side effects of the drugs that are used to treat clinically significant PDA in the newborn, their doses, duration of the treatment, and how many courses of medication should be performed; and also there are different opinions regarding the timing of surgical intervention [1, 11]. Considering the fact that the side effects of the drugs are more prominent in VLBWPN, we advocate early surgical ligation may be a better approach. The results from previous studies by Grosfeld et al. and Cassady et al. support our opinion, in which it was underlined that early surgery should be the treatment of choice in very low birth weight premature babies [12, 13]. Similarly, in their 2009 study conducted with 41 premature children, Ko et al. suggested early surgical ligation of PDA without medical treatment in VLBWPN should be done, in order to reduce the complications of PDA and medications [4]. In another ongoing study of us, we aim to compare the VLBWPN that underwent early PDA ligation without prior medical treatment, that took medical treatment successfully, that underwent surgical ligation after unsuccessful medical treatment, and that underwent surgical ligation after reopening of the medically closed PDA.

Mortality and morbidity rates of surgical closure of PDA in premature babies are low [14]. Mortier et al. reported their experience in 33 premature infants with PDA who have been operated in their NICU(neonate intensive care unit) over a six-year period and they observed no operative or immediate postoperative deaths and reported hospital mortality was 6 % [15]. Likewise Ko et al. showed no mortality in 42 VLBWPN [4]. No surgical mortality occurred in our patients. Surgical ligation is accomplished through posterolateral thoracotomy. This method can lead to serious morbidity and mortality such as pneumothorax, chilothorax, infections, paralysis of laryngeal nerve, respiratory failure, hemodynamic instability, bronchopulmonary dysplasia, retinopathy of the premature, and death [16]. No surgical complication occurred during our study, except pneumothorax in one case.

Transcatheter treatment of patent ductus arteriosus can be used for patients of varying ages, except small babies under 5 kg and small PDA [17]. Despite the advancement in the procedure, there are many problems during performance of transcatheter PDA closure in infants: relatively large sheath size for small vessels, stiffness of the delivery system with resultant hemodynamic instability during device deployment, risk of protrusion of the device into the aorta or pulmonary artery, poor anchoring or stability within the PDA, and difficult retrievability [18, 19].

Currently babies can be safely operated in NICU environment. The operations include gastrostomy, PDA ligation, tracheostomy, repairment of trachea-esophageal fistulae, laparotomies, repairment of abdominal wall defects, stroma closure, posthemorrhagic hydrocephalus surgery, and several urinary interventions [20]. The results of our study supports that PDA ligation can be successfully performed in NICUs. One important issue is the experience of and harmony among the team members, namely neonatologist, anesthesiologist, surgical team members, nurses, and surgical technologists. As the experience and harmony levels promote, better success rates and shorter operation times can be achieved. Our first bed-side PDA ligation performance took 63 min from initial positioning of the baby to the end of the operation, while the same window of time decreased to 30 min in our last case. Another important point is the physical conditions of NICU. The side walls and roof of the infant incubators must be completely removable. In our opinion, the operations that were performed in NICU after transferring the baby to an open bed because of the incompatibility of the infant incubator should not be deemed as bed-side, because in those cases the potential risks of transportation can’t be eliminated completely. Our operations were performed with ease in Giraffe® Incubator by General Electric’s Company fully-removable incubators. Also led lights, radiant heater, led head-lamps, neonatal respirator, suction system, multiparameter monitor (with ECG, pulse oximeter, invasive/noninvasive arterial monitorization, central venous pressure monitorization, and temperature monitorization, etc.), appropriate surgical tools for VLBW premature infants, nitrogen dioxide cylinder, and sevoflurane must be readily available in the NICU. Our bed-side PDA ligation interventions in LBW premature babies reduced the risks of transportation. Several possible adverse events during transportation such as hypothermia, trauma, dislocation of the tracheal tube, dislocation of the vascular lines, and hemodynamic instability may lead to an increased morbidity in the VLBW premature. In all of our cases, PDA was successfully closed bed-side through posterolateral thoracotomy under general anesthesia in NICU, with no surgical mortality.

## Conclusion

Bedside PDA ligation in the NICU is a safe method for VLBWPN with physiologically significant PDA. According to the results of our study and our clinical experience, early surgical ligation shall be the treatment of choice in VLBWPN with PDA, in order to minimize the possible complications of indomethacin and ibuprofen medications, prolonged intubation, and PDA itself; although, medical closure with these drugs was attempted in all of the cases in our study. In addition, we would like to underline as a conclusion that, whenever appropriate NICU conditions and an experienced team is available, bedside PDA ligation is definitely the best choice for the treatment of whether VLBWPN, or mature neonates, in accordance with the results of our studies and the current literature.

## Abbreviations

NICU: Neonate intensive care unit
PDA: Patent ductus arteriosus
VLBWPN: Very-low-birth-weight premature neonates
